# Efficacy of Experimental Mouth Rinses on Caries-Related Biofilms *in vitro*

**DOI:** 10.3389/froh.2021.676028

**Published:** 2021-07-14

**Authors:** Josiana Steiger, Olivier Braissant, Tuomas Waltimo, Monika Astasov-Frauenhoffer

**Affiliations:** ^1^Clinic for Oral Health & Medicine, University Center for Dental Medicine Basel UZB University of Basel, Basel, Switzerland; ^2^Department of Biomedical Engineering (DBE), Center of Biomechanics and Biocalorimetry, University of Basel, Allschwil, Switzerland; ^3^Department Research, University Center for Dental Medicine Basel UZB University of Basel, Basel, Switzerland

**Keywords:** caries, biofilm, antimicrobial, isothermal microcalorimetry, amine fluoride, stannous fluoride, PEG-3 tallow aminopropylamine

## Abstract

This study assessed the efficacy of tin and Polyethylenglycol (PEG-3) tallow aminopropylamine in different concentrations on *Streptococcus mutans (S. mutans)* biofilms to establish a new screening process for different antimicrobial agents and to gain more information on the antibacterial effects of these agents on cariogenic biofilms. Isothermal microcalorimetry (IMC) was used to determine differences in two growth parameters: lag time and growth rate; additionally, reduction in active biofilms was calculated. Experimental mouth rinses with 400 and 800 ppm tin derived from stannous fluoride (SnF_2_) revealed results (43.4 and 49.9% active biofilm reduction, respectively) similar to meridol mouth rinse (400 ppm tin combined with 1,567 ppm PEG-3 tallow aminopropylamine; 55.3% active biofilm reduction) (*p* > 0.05), while no growth of *S. mutans* biofilms was detected during 72 h for samples treated with an experimental rinse containing 1,600 ppm tin (100% active biofilm reduction). Only the highest concentration (12,536 ppm) of rinses containing PEG-3 tallow aminopropylamine derived from amine fluoride (AmF) revealed comparable results to meridol (57.5% reduction in active biofilm). Lower concentrations of PEG-3 tallow aminopropylamine showed reductions of 16.9% for 3,134 ppm and 33.5% for 6,268 ppm. Maximum growth rate was significantly lower for all the samples containing SnF_2_ than for the samples containing control biofilms (*p* < 0.05); no differences were found between the control and all the PEG-3 tallow aminopropylamine (*p* > 0.05). The growth parameters showed high reproducibility rates within the treated groups of biofilms and for the controls; thus, the screening method provided reliable results.

## Introduction

The oral microflora represents the complex community and cohabits of up to 1,000 different bacterial species [1–4]. Most of the microorganisms favor colonization and adherence on surfaces forming biofilms whenever possible [5–7]. Caries is still a frequently occurring oral health issue in the Global Burden of Disease 2015 study [8, 9]. It has been known for a long time that the group of mutans streptococci and especially, *Streptococcus mutans* is the specific pathogen that causes caries. However, more important than one key pathogen is the environment that influences the homeostasis of the biofilm. In healthy conditions, demineralization and remineralization of the tooth surface are in balance and no irreversible defect occurs. Through the consumption of a carbohydrate-rich diet and/or too little neutralizing saliva, the environment can become more acidic due to the metabolic products of the bacteria. Thus, the balance is not maintained and aciduric and acidogenic bacteria, such as *S. mutans* and *Lactobacilli*, gain predominance and accelerate the demineralization, leading to dysbiosis of the microbiota [7, 10–12].

Associations between oral and general health make oral hygiene indispensable. Although tooth brushing is very important to mechanically remove the biofilm, antimicrobial agents used in oral care products like mouth rinses are also very important to protect against the progression of dental caries. Fluorides, in addition to chlorhexidine, cetylpyridinium chloride (CPC), peroxide/perborates, essential oils (EOs), triclosan, delmopinol, and sanguinarine, are occurring agents in mouthwashes [13, 14].

This study focused on the inorganic compound tin derived from stannous fluoride (SnF_2_), with its antimicrobial and cariostatic characteristics, and the organic compound of amine fluoride (AmF) PEG-3 tallow aminopropylamine, which can reduce the adherence of bacteria leading to plaque formation and may also influence the diffusion through the caries-affected enamel [15]. Different studies have shown that a *S. mutans* biofilm treated with AmF leads to a reduction in growth [16], while the antibacterial properties of PEG-3 tallow aminopropylamine, known to act as a surfactant in different kinds of toothpaste, have been assessed to reveal a reduction in vitality for periopathogenic and cariogenic species [17].

Isothermal microcalorimetry (IMC) is a highly sensitive culture-based method that allows the measurement of heat generated by all the microbial activities within the biofilm without disturbing its structure. It allows bacteria to replicate on a suitable culture medium, resulting in an exponential increase in the heat production rate that can be recorded in real time by IMC (i.e., heat flow curve). The main advantage of this method in biofilm research is the opportunity to study them as entities as shown previously [18]. Over the last decade, the method has become more widely used in antimicrobial testing in various fields of medicine [19–22].

This study aimed to use IMC to assess the efficacy of tin and PEG-3 tallow aminopropylamine in different concentrations on *S. mutans* biofilms to establish a new screening process for different antimicrobial agents and to gain more information on the antibacterial effects of these agents on cariogenic biofilms; the antibacterial effect of fluoride was not considered. The null hypothesis was that both antimicrobial agents reveal similar efficacy.

## Materials and Methods

### Biofilm Formation and Treatment

Ten μl of *S. mutans* (ATCC® 25175) stock solution was spread on a Columbia blood agar plate (BBL Columbia Agar Base; BD, Allschwil, Switzerland) and incubated for 48 h at 37°C. Thereafter, one colony was picked and added to 25 ml of Todd Hewitt (TH) media (Bacto Todd Hewitt broth; BD, Allschwil, Switzerland) supplemented with 0.5% sucrose [D(+) sucrose; Fluka, Buchs, Switzerland]. The culture was incubated for 22 h at 37°C. Then, the bacteria were harvested by centrifugation (8,500 rpm, 5 min, RT; Sigma 4-16KS, Kuhner, Basel, Switzerland) and resuspended in simulated body fluid (SBF; [23]) supplemented with 10% TH medium and 1% sucrose. Meanwhile, 5 mm glass disks (Biosystems Switzerland AG, Muttenz, Switzerland) were coated with sterile pooled saliva mixture for 15 min (described in detail in Astasov-Frauenhoffer et al. [24]) and placed in 24-well plates (Sarstedt AG, Sevelen, Switzerland), 1 ml of bacterial suspension and 0.5 ml of TH medium were added to each well, and the disks were incubated for 24 h at 37°C.

After 24 h, the disks with biofilms were dipped x3 in 0.9% NaCl (Merck, Zug, Switzerland) and then placed for 30 s in 50% v/v mouth rinse solutions (Table 1) and placed in 0.9% NaCl. Biofilms exposed to 0.9% NaCl for 30 s served as untreated growth controls. Each set consisted of five replicates, and experiments were repeated three times altogether.

**Table 1:** One marketed mouth rinse and six experimental mouth rinses used in this study based on their major antimicrobial agent and their concentrations.

**Mouth rinse type**	**Marketed rinse**	**Experimental rinses**					
	**Meridol**	**1**	**2**	**3**	**4**	**5**	**6**
Main antibacterial agent	Tin/PEG-3 tallow aminopropylamine	Tin	Tin	Tin	PEG-3 tallow aminopropylamine	PEG-3 tallow aminopropylamine	PEG-3 tallow aminopropylamine
Calculated Concentration (ppm)	400/1,567	400	800	1,600	3,134	6,268	12,536

### Isothermal Microcalorimetry

Columbia blood agar was prepared, and treated biofilms and untreated controls were placed on the agar with the biofilm facing the agar to monitor the microbial metabolic activity left in the samples. The IMC ampoules were closed in aerobic conditions and placed in TAM 48 instrument (TA Instruments; New Castle, Delaware, USA) where the metabolic activity of the biofilms was recorded at 37°C for up to 72 h. Samples that now reveal any growth during that time period were also assessed over 7 days.

The heat flow data obtained over time were analyzed for growth rate (1/h) and lag time (h) by fitting the heat over time curve (i.e., resulting from the integration of the heat flow curve) with the Gompertz equation with the “grofit” package in R statistical software (R Foundation for Statistical Computing, Vienna, Austria) as described earlier [18].

Taking into consideration the average growth rate of biofilm and the correspondence of increase in lag time [roughly a biofilm doubling time] to a x2 reduction in the bacterial population [25], the reduction in the biofilm population was estimated by using the following approach (1):


(1) $$\begin{array}{r} \text{Biofilm reduction }(\%)=\text{100}-({\text{100/2}}^{\wedge }((\text{lag }{\text{time}}_{\text{treated sample}}\\ -\text{lag }{\text{time}}_{\text{control}})\text{/}(\text{ln}(\text{2})\text{/growth }{\text{rate}}_{\text{control}}))). \end{array}$$

### Statistical Analysis

The Shapiro–Wilk test for normality was applied to the samples, differences between mouth rinses and untreated controls were assessed by using Student's *t-*test with significance set to *p* < 0.05, and differences between the different concentrations were assessed by a one-way ANOVA using GraphPad Prism (version 7.00 for Mac, GraphPad Software, La Jolla, California, USA, www.graphpad.com).

## Results

Three different parameters (lag time and maximum growth rate, and reduction in active biofilm) were assessed from the IMC data, and they are presented in Table 2.

**Table 2:** Three different parameters were assessed from IMC data: lag time in h, maximum growth rate in 1/h, and reduction in active biofilm in % compared to untreated control biofilm.

**Parameter**	**Untreated control**	**Marketed rinse**	**Experimental rinses**					
		**Meridol**	**1**	**2**	**3**	**4**	**5**	**6**
Lag time (h)	8.55 ± 0.4	18.9 ± 2.1^**^	15.9 ± 1.0^**^	17.4 ± 1.5^**^	>72^**^	10.9 ± 0.6^**^	13.8 ± 1.1^**^	19.5 ± 2.7^**^
Maximum growth rate (1/h)	0.078 ± 0.004	0.066 ± 0.003^**^	0.065 ± 0.003^**^	0.064 ± 0.003^**^	0^**^	0.074 ± 0.006	0.076 ± 0.006	0.072 ± 0.007
Reduction of active biofilm (%)	N/A	55.3 ± 7.6	43.4 ± 4.4^**^	49.9 ± 5.6^*^	> 99^**^	16.9 ± 4.2^**^	33.5 ± 5.5^**^	57.5 ± 8.1

*Seven rinses were examined for their antimicrobial properties containing either tin derived from stannous fluoride (SnF_2_) and/or PEG-3 tallow aminopropylamine as noted in Table 1. Statistical differences were marked with*

** *p < 0.0001*,

* *p < 0.05*.

Dose-dependent reduction in active biofilms was detected for samples treated with increasing concentrations of PEG-3 tallow aminopropylamine; however, no differences (*p* > 0.05) were measured for maximum growth rate at the same time, while statistical differences in LT (*p* < 0.05) were detected. Using concentrations of 400 and 800 ppm tin revealed results similar to meridol mouth rinse (although statistically seen as there were differences found, *p* < 0.05), while growth was inhibited during the whole detection period of 72 h when samples were treated with an experimental rinse containing 1,600 ppm tin. Interestingly, when assessing the reduction percentage in active biofilm, only the highest concentration (12,536 ppm) of PEG-3 tallow aminopropylamine revealed comparable results to meridol (55.3% reduction in active biofilm) and rinses based on SnF_2_ (43.4% for 400 ppm tin, 49.9% 800 ppm tin, and >99% of active biofilm reduction for 1,600 ppm tin) reaching to the average reduction of about 57.5%. Lower concentrations of PEG-3 tallow aminopropylamine showed reductions of 16.9% for 3,134 ppm and 33.5% for 6,268 ppm. Maximum growth rate was significantly lower for all the samples containing SnF_2_ than for the samples containing control biofilms (*p* < 0.05); no differences were found between the control and all the PEG-3 tallow aminopropylamine (*p* > 0.05). Samples treated with experimental rinse 3 also revealed no growth after 7 days, allowing the assumption that >99% of the active biofilms was eradicated.

The parameters lag time and growth rate both showed high reproducibility rates within the treated groups of biofilms and for the controls; thus, the screening method provided reliable results.

## Discussion

The efficacy of different mouth rinses on plaque control has been analyzed in many studies. Fluorides, which are mainly used to reinforce demineralization, have also shown antimicrobial properties [26] and can affect, for example, *S. mutans* through various mechanisms [27–29]. This antibacterial effect of SnF_2_ in cariogenic biofilms can be attributed to the ability of stannous ion to reduce bacterial acidogenicity and glucan production [30], while AmF is shown to inhibit the APTase of *S. mutans* [31]. Additionally, there is not only the efficacy that makes such fluoride formulations (e.g., SnF_2_/AmF) so attractive but also there are fewer side effects like cytotoxicity and irritations than in mouth rinses with essential oils or chlorhexidine [32]. Furthermore, regarding resistance development in *S. mutans* strains, AmF/SnF_2_ can be used for long term in contrast to chlorhexidine, which should be used only for a limited time [33].

However, the major antimicrobial effect of mouth rinses is achieved through various other agents added to the rinses. Thus, this study examined the susceptibility of *S. mutans* biofilms to different experimental mouth rinses with various concentrations of either tin derived from SnF_2_ (400, 800, and 1,600 ppm) or PEG-3 tallow aminopropylamine (3,134, 6,268, 12,536 ppm) and meridol containing both (400 ppm tin + 1,567 ppm PEG-3 tallow aminopropylamine). The motivation to use these agents lies within the fact that, although both are widely used in new oral care products, the contribution to the antimicrobial effect of single agents within these products is not always clearly defined. Additionally, screening for the best possible concentration allows designing of more efficient rinses based on these data.

The combination of 400 ppm tin derived from SnF_2_ and 1,567 ppm PEG-3 tallow aminopropylamine derived from AmF (meridol) is showing comparable results (average of 55.3% active biofilm reduction) to the experimental mouth rinse containing 12,536 ppm PEG-3 tallow aminopropylamine, reaching a reduction in active biofilm by 57.5%. Rinses containing AmF/SnF_2_ have shown antiplaque and also antibacterial effects [34–41]. Furthermore, this formulation is able to influence the streptococcus abundance and lactate production [42] by inhibiting the acid production to the same extent as chlorhexidine [43]. The study by Meurman et al. demonstrated the sensitivity of *S. mutans* to an AmF/SnF_2_ solution *in vitro*. Regarding the topic of cariology, it is mentioned that there are, maybe, some advantages in the usage of SnF_2_/AmF solution compared to chlorhexidine [44].

Additionally, we also found that an increasing concentration of tin (400, 800, and 1,600 ppm) leads to a decrease in active biofilm and a prolonged lag time. Thus, tin has also an antibacterial effect that is dose dependent. However, the relation between the concentration and the reduction in active biofilm is not proportional. Regarding the active biofilm reduction, more than 800 ppm tin is needed to achieve approximately comparable effects to the SnF_2_/AmF solution. However, the efficacy of rinse 3 containing tin at a concentration of 1,600 ppm was inhibiting the growth of biofilms throughout the detection period; thus, we cannot say whether the whole biofilm was killed, or it would have shown delayed growth after the 72 h. Therefore, for obtaining results for higher antimicrobial concentrations, the metabolic patterns of IMC should be followed for prolonged periods of time. The reduction is achieved through both bactericidal effects as the lag time is prolonged for these samples in comparison with untreated control; thus, a larger proportion of cells was killed in the beginning, as well as bacteriostatic effect as the growth rate is decreased, which means that the bacterial growth was impaired throughout the measurement. This was only to be seen in the treated samples that contained SnF_2_; the effect of PEG-3 tallow aminopropylamine seems to be only bactericidal as the growth rate is not affected and stays similar to the untreated control biofilm.

Svanberg and Rölla have shown that *S. mutans* counts in plaque and in saliva can be reduced through rinsing with SnF_2_ and have suspected that this is a possible reason for a decrease in the occurrence of caries. Additionally, one of the tested concentrations of SnF_2_ (0.2 and 0.4%) was comparable to our tested concentration in rinse 3, 1,600 ppm (0.22%) [45]. The formulation of SnF_2_ in water-based solutions is challenging because of the diminished stability [13, 46]. In the formulation SnF_2_ together with AmF (meridol), the positive properties of the SnF_2_ are stabilized. Synergistic effects led to a positive effect in caries prevention and periodontal health through plaque reduction [13, 47]. The increasing concentration of PEG-3 aminopropylamine shows a proportional ratio to the reduction in active bacteria biofilm. At concentrations of 12,536 ppm, the antibacterial effect is even somewhat higher than in the meridol.

An advantage of using IMC is fast and reliable screening that generates a real-time signal with high sensitivity of the produced heat by microorganisms, and unlike spectrophotometric analyses, it is not affected by the possible turbidity of the solution caused by antimicrobial agents. In addition, the non-destructive property enables the efficient use of this method for biofilms, without disturbing their structure and function [18, 25, 48], and measuring the extent of antimicrobial effect of solutions on biofilms. However, the biggest limitation of the study is the unknown efficacy of rinses while planning the experiments and being unable to predict how long the detection time should be. Nevertheless, it can be easily overcome by running a test experiment to see how long the samples need for their heat flow curve to return to baseline. Additionally, the study uses water for the dilution of rinses; thus, no precipitation should happen while doing so. Also, here, it can be easily adapted to another solution as long as the rinsing step is carried out with saline before placing the biofilms in the IMC ampoule.

In conclusion, the study revealed that the effect of SnF_2_, in combination with AmF, is enhanced and mouth rinse also is effective in caries-related biofilm reduction when concentrations of these two agents are low. To achieve a similar effect with SnF_2_ or PEG-3 aminopropylamine alone, higher concentrations are needed, which, however, might lead to resistance development over time. Thus, the choice of mouth rinse has to be carefully considered based on whether short- or long-term use is planned. Additionally, the study also shows that IMC is a reliable tool to use for future screenings on the efficacy of antimicrobial agents on biofilms as shown earlier [18, 49], providing highly reproducible results throughout the experiment despite possible biofilm variability. However, it is important to adjust the screening period to the efficacy of the rinses in order to obtain a reliable metabolic pattern to assess the growth parameters.

Furthermore, this study introduces a model biofilm combined with a reliable screening method for all future investigations in which the antimicrobial effect of solutions on cariogenic biofilms could be easily assessed; with the big advantage in comparison with many other studies, the biofilm stays intact throughout the whole process and the effect of planktonic cells can be neglected. Thus, various antimicrobial agents or optimization of concentrations and/or combinations of such agents and proportions of concentrations of tin and PEG-3 aminopropylamine can be studied further for their resistance development using this model.

## Data Availability Statement

The raw data supporting the conclusions of this article will be made available by the authors, without undue reservation.

## Author Contributions

OB, TW, and MA-F: conceptualization. OB and MA-F: methodology and software. JS, OB, TW, and MA-F: validation, data curation, and writing—review and editing. MA-F: formal analysis, project administration, and funding acquisition. JS and MA-F: investigation, writing—original draft preparation, and visualization. TW: resources. TW and MA-F: supervision. All authors have read and agreed to the published version of the manuscript.

## Conflict of Interest

This research was funded by COLGATE-PALMOLIVE EUROPE sàrl. The funders had no role in the design of the study; in the collection, analyses, or interpretation of data; in the writing of the manuscript, or in the decision to publish the results. The authors declare that the research was conducted in the absence of any commercial or financial relationships that could be construed as a potential conflict of interest.
